# Professionally Created Content Related to HPV Vaccination on TikTok

**DOI:** 10.3389/fdgth.2022.888302

**Published:** 2022-06-28

**Authors:** Corey H. Basch, Grace C. Hillyer, Erin T. Jacques

**Affiliations:** ^1^Department of Public Health, William Paterson University, Wayne, NJ, United States; ^2^Department of Epidemiology, Mailman School of Public Health, Columbia University, New York City, NY, United States; ^3^Department of Health and Human Performance, York College, Jamaica, NY, United States

**Keywords:** TikTok, Human Papillomavirus (HPV), vaccination, professional—patient relations, social media

## Abstract

Despite the protective abilities of the HPV vaccine, roughly half of adolescents in the United States have not completed the recommended HPV vaccine series. Professionals have taken to using social media platforms to encourage health behaviors such as receipt of the HPV vaccine. As such, the purpose of this study was to identify content created by professionals related to HPV on TikTok. This descriptive, cross-sectional study was conducted in January 2022 using the hashtag #HPV Vaccine to examine the 100 English language videos created by people who claimed to be health professionals. In addition to capturing metadata, each videos' content and subsequent comments were coded. Overall, 75.0% of the videos mentioned HPV-related cancer but few discussed vaccination as a cancer preventive measure (40.0%). More than half (52.0%) of the comments were neutral in tone and most focused on cancer (54.0%), alternative medicine (58.0%), and general questions about vaccination (62.0%). Comments about videos with greater numbers of “likes” more often mentioned cancer (85.0% vs. 46.3%, *p* = 0.002), the age at which to get vaccinated (70.0% vs. 41.3%, *p* = 0.02) and more frequently posed questions about vaccination (80.0% vs. 41.3%, *p* = 0.002) and cost and insurance coverage of vaccination (35.0% vs. 11.3%, *p* = 0.02) compared to videos with fewer “likes.” The power of provider information is paramount with HPV vaccine uptake. As providers increasingly create health messages on platforms such as TikTok, it is important that they remain aware of the potential for opposing or non-factual discourse.

## Introduction

Human Papillomavirus (HPV) is the most frequently diagnosed viral infection of the reproductive tract (1) and is the most common sexually transmitted infection in the United States (U.S.) (2). In 2018, it was estimated that there were 43 million active HPV infections in the U.S. with an additional 13 million new cases (2). There are more than 100 strains of HPV, although not all strains are problematic to one's health (3). Types 6, 11, 16 and/or 18 are most commonly linked to health problems which range in severity from genital warts to cancer of the cervix, vulva, vagina, penis, anus, and oropharynx (3, 4). Over 95% of cervical cancer cases are due to HPV (1).

The United States Food and Drug Administration (FDA) has approved vaccines that prevent cervical cancer and other diseases caused by the more virulent types of HPV (3). Currently, there are three vaccines that prevent disease-causing types of HPV infection licensed in the U.S.—Gardasil, Gardasil 9^®^, and Cervarix but, of these, Gardasil^®^ 9 is the only HPV vaccine used in the U.S. (5). It prevents infection from the 9 HPV types associated with both genital warts and HPV-associated cancers, particularly cervical cancer (5). The Advisory Committee on Immunization Practices (ACIP) recommends that 9–14 year old teens receive two doses of the HPV vaccine within a period of 6 to 12 months and teens and young adults 15–26 years receive three doses over a 6-month period (6). Despite the protective abilities of the HPV vaccine, the Centers for Disease Control and Prevention (CDC) estimates that roughly half of U.S. adolescents have not completed the recommended HPV vaccine series (7).

There are several factors which influence uptake of the HPV. Vaccine hesitancy is a major issue when it comes to receipt of the HPV vaccine. As this is a vaccine distributed to adolescents, it would be remiss not to consider parental attitudes in decision making (8–11). Parents who have distrust toward the medical establishment (12, 13), reported fear of condoning sexual activity (14), and find a lack of necessity (15) are all less likely to choose to vaccinate their children. Further, information about HPV vaccination can be difficult to read and thus could influence choices, especially in populations with low health literacy (16). Vaccine hesitancy is not exclusively the result of not wanting the vaccine. Lack of access to healthcare (17–19) is a major factor in uptake of vaccines, as are safety concerns.

An equally important consideration is the role that adolescents themselves play in influencing their vaccine uptake. Some states are making concerted efforts to allow for adolescents to receive the vaccine without parental consent (11, 20, 21). Research indicates that adolescents' alone have marginal willingness to receive the HPV vaccines, which may increase as adolescents cross the threshold into adulthood (18 years and older) (22). Those who engage with HPV vaccine information on social media may be exposed to unfavorable, inaccurate, and/or incomplete information (23).

The influx of health information on the internet has been regarded by researchers as paradoxical: being both highly beneficial yet potentially hazardous at the same time. While the internet presents an opportunity for the general public to gather user-friendly information that is comprehensible and readily accessible (24), evidence-based information and disinformation share the same space. Among the internet channels, social media platforms such as TikTok are quickly becoming an effective communication medium in healthcare (25). During the COVID-19 pandemic, TikTok became the most popular web domain with an impressive 1 billion monthly users (26, 27). The most common users of TikTok are those aged 12–17 years (28) and females (29, 30). Researchers have indicated that consumer-driven content on TikTok can complicate medical recommendations for vaccines, as content can contain misinformation and disinformation, which can have harmful consequences (31).

However, increasingly, health professionals are creating TikTok health-related videos that use humor, trendy songs, and self-criticism to connect with young audiences (23). Adolescents can consent and obtain the HPV vaccine on their own. Because they are great consumers of social media, examining TikTok videos helps us to understand what information they are getting and allows us to better understand their vaccination behavior with the hopes of positively influencing that behavior and encouraging vaccination.

Many manuscripts in the published literature document that HPV content is prevalent on social media. Research has identified specific platforms such as Instagram (32–34), YouTube (35–37), Twitter (38), and Facebook (12) but we were only able to identify a single paper that examined HPV vaccine content on TikTok but was not limited to professionals (39). Examination of professional videos can help guide future health promotion efforts as this population begins to deliver more information on social media. To date, we did not identify any studies that exclusively examined content created by professionals related to HPV on TikTok. Hence, that is the purpose of this study.

## Methods

This descriptive, cross-sectional study was conducted in January 2022. Using TikTok's discover search feature, the hashtag #HPV Vaccine was identified as the most relevant hashtag with 6.4M views. The first 100 videos created by people who claimed to be health professionals were selected for inclusion. This methodology has been used successfully in other studies (31). Video creators self-reported their health professional status by communicating it orally in their videos and/or in their written profile descriptions. These professionals included medical doctors, nurses, health educators, auxiliary health workers, and other medical staff. Health professionals' videos were coded for their format, content and comments. In assessing video format, it was determined if the video used humor and/or dance. The video content demonstrates the types of information health professionals share about HPV. In contrast, the comments give insight into how users perceive and react to the videos. The content and comments collectively tell a larger story about thoughts related to HPV vaccine uptake. The videos that were not in English, those with ambiguous content, and those created by persons other than self-identified health professionals were excluded.

One researcher (ETJ) recorded the videos' metadata and coded the videos' content and comments and watched all videos. The coding categories used to describe the video content included: pro, con, and neutral sentiments about the vaccine; the mention of: HPV-related cancer; HPV prevention; HPV testing/screening; HPV transmission; HPV infection risk factors; HPV vaccines are safe/unsafe; and side effects of HPV vaccine. Categories used to organize the comments from the videos included: pro-vaccine/anti-vaccine/ neutral HPV vaccine messages; mention of cancer, age for the vaccine, males being vaccinated, cost/insurance, fertility concerns, use of alternative medicine, general vaccination questions, and sharing of stories related to HPV vaccination. A second researcher (CHB) coded a random 10% sample to determine inter-rater reliability, which was high based on Cohen's *kappa* coefficient (0.95).

We performed a descriptive analysis of the characteristics of the 100 TikTok videos that included frequency distributions of dichotomous variables and calculated means, standard deviations, and ranges for continuous variables. To test for differences between videos with comparatively low numbers of “likes” and those with high numbers of “likes”, we dichotomized the total number of “likes” at the mean and performed univariable analyses using chi square test and Analysis of Variance (ANOVA) for dichotomous and continuous variables, respectively. *P*-values <0.05 were considered statistically significant. All analyses were conducted using SPSS version 28 (40). Since the study did not require the participation of human subjects, William Paterson University's Institutional Review Board determined the study did meet the criteria for review.

## Results

Of the 100 TikTok videos reviewed, the majority were posted in 2021 (66.0%) with a mean of 3,554 [SD 9,046] “likes” (Table 1). Only 15% of the videos used humor, while 54% used music. All videos in this sample (100%) were pro-vaccination. Overall, 75.0% of the videos mentioned HPV-related cancer. Fewer videos discussed vaccination specifically as a cancer preventive measure (40.0%), HPV screening and testing (27.0%), how HPV is transmitted (36.0%), risk factors associated with HPV infection (4.0%), and that the vaccine is considered safe (10.0%). More than half (52.0%) of the comments were neutral in tone, that is, neither pro- nor anti-vaccination and most focused on cancer (54.0%), alternative medicine (58.0%), and general questions about vaccination (62.0%). When looking at the differences between videos with relatively high “likes” (≥3,554) compared to those with lower numbers of “likes” (<3,554), more recent videos (posted in 2021) more often had fewer “likes” (70.0% vs. 50.0%, *p* = 0.02); no differences with regard to video format and content was observed. Comments about videos with greater numbers of “likes” more often mentioned cancer (85.0% vs. 46.3%, *p* = 0.002), the age at which to get vaccinated (70.0% vs. 41.3%, *p* = 0.02) and more frequently posed questions about vaccination (80.0% vs. 41.3%, *p* = 0.002) and cost and insurance coverage of vaccination (35.0% vs. 11.3%, *p* = 0.02) compared to videos with fewer “likes” (Table 2).

**Table 1:** Characteristics of TikTok videos related to HPV vaccination (*n* = 100) by number of “likes”, dichotomized at the mean.

	**Total**	**Likes lower than the mean (*n =* 80)**	**Likes equal to or greater than the mean (*n =* 20)**	***P*-value**
**Video characteristics**				
Year posted				0.02
2019	2 (2.0)	0 (0.0)	2 (10.0)	
2020	32 (32.0)	24 (30.0)	8 (40.0)	
2021	66 (66.0)	56 (70.0)	10 (50.0)	
Comments				<0.001
Mean [SD]	129 [282]	60 [123]	406 [501]	
Range	0–2,285	0–889	0–2,285	
Forwards				<0.001
Mean [SD]	95 [252]	25 [46]	375 [467]	
Range	0–1,946	0–238	52–1,946	
**Video format**				
Uses humor				0.08
Yes	15 (15.0)	9 (11.3)	6 (30.0)	
No	85 (85.0)	71 (88.8)	14 (70.0)	
Uses music				0.27
Yes	54 (54.0)	41 (51.2)	13 (65.0)	
No	46 (46.0)	39 (48.8)	7 (35.0)	
**Video content**				
Video creator point of view				–
Pro vaccination	100 (100.0)	80 (80.0)	20 (20.0)	
Anti-vaccination	0 (0.0)	0 (0.0)	0 (0.0)	
Pro and anti-vaccination	0 (0.0)	0 (0.0)	0 (0.0)	
Neutral (neither pro nor con)	0 (0.0)	0 (0.0)	0 (0.0)	
Mentions HPV-related cancer				0.25
Yes	75 (75.0)	62 (77.5)	13 (65.0)	
No	25 (25.0)	18 (22.5)	7 (35.0)	
Mentions HPV prevention				0.61
Yes	40 (40.0)	31 (38.8)	9 (45.0)	
No	60 (60.0)	49 (51.3)	11 (55.0)	
Mentions HPV testing/screening				0.18
Yes	27 (27.0)	34 (30.0)	3 (15.0)	
No	73 (73.0)	56 (70.0)	17 (85.0)	
Mentions HPV transmission				0.92
Yes	36 (36.0)	29 (36.3)	7 (35.0)	
No	64 (64.0)	51 (63.7)	13 (65.0)	
Mentions HPV infection risk factors				0.70
Yes	4 (4.0)	4 (5.0)	0 (0.0)	
No	96 (96.0)	76 (95.0)	20 (100.0)	
Mentions vaccine as safe				0.21
Yes	10 (10.0)	10 (12.5)	0 (0.0)	
No	90 (90.0)	70 (87.5)	20 (100.0)	
Mentions vaccines as unsafe				–
Yes	0 (0.0)	–	–	
No	100 (100.0)	–	–	
Mentions vaccine side effects				0.57
Yes	5 (5.0)	5 (6.3)	0 (0.0)	
No	95 (95.0)	75 (93.8)	20 (100.0)	

**Table 2:** Comments about TikTok video related to HPV vaccination (*n* = 100) by number of “likes”, dichotomized at the mean.

	**Total**	**Likes lower than the mean (*n =* 80)**	**Likes equal to or greater than the mean (*n =* 20)**	***P*-value**
**Comment characteristics**				
Viewer reactions				0.15
Pro vaccination	16 (16.0)	13 (16.3)	3 (15.0)	
Anti-vaccination	11 (11.0)	10 (12.5)	1 (5.0)	
Pro and anti-vaccination	21 (21.0)	13 (16.3)	8 (40.0)	
Neutral (neither pro nor con)	52 (52.0)	44 (55.0)	8 (40.0)	
**Comment content**				
Mentions HPV-related cancer				0.002
Yes	54 (54.0)	37 (46.3)	17 (85.0)	
No	46 (46.0)	43 (53.8)	3 (15.0)	
Mentions age for vaccine				0.02
Yes	47 (47.0)	33 (41.3)	14 (70.0)	
No	53 (53.0)	47 (58.8)	6 (30.0)	
Mentions awareness of males getting vaccine				0.56
Yes	25 (25.0)	19 (23.8)	6 (30.0)	
No	75 (75.0)	61 (76.3)	14 (70.0)	
Mentions cost/insurance				0.02
Yes	16 (16.0)	9 (11.3)	7 (35.0)	
No	84 (84.0)	71 (88.8)	13 (65.0)	
Mentions fertility concerns				0.30
Yes	11 (11.0)	7 (8.8)	4 (20.0)	
No	89 (89.0)	73 (91.3)	16 (80.0)	
Mentions alternative medicine				0.84
Yes	58 (58.0)	46 (57.5)	12 (60.0)	
No	42 (42.0)	34 (42.5)	8 (40.0)	
General vaccine questions				0.18
Yes	62 (62.0)	47 (58.8)	15 (75.0)	
No	38 (38.0)	33 (41.3)	5 (25.0)	
Shares stories or experience with HPV				0.002
Yes	49 (49.0)	33 (41.3)	16 (80.0)	
No	51 (51.0)	47 (58.8)	4 (20.0)	

## Discussion

In a TikTok study of both professional and non-professional videos that varied in pro/anti vaccine stance, it was determined that cancer and prevention messaging on HPV was more prevalent among pro-vaccine videos (39). Our findings are in concert with these in that all videos included in our sample were created by professionals and were pro-vaccine. We found that the frequency with which HPV-related cancer was mentioned was higher, while specifically mentioning prevention was lower in our sample of TikTok videos.

Our findings demonstrated that most of the professionally-made videos in this sample discussed HPV-related cancers. Studies have focused on the effects of framing messages related to HPV around cancer prevention risk and/or prevention (13, 41, 42) rather than on the sexually transmitted nature of the infection. User comments were predominantly neutral in their position about HPV vaccination uptake. One study of comments on HPV-related YouTube videos showed that comments tended to have anti-vaccine sentiment, but this study was not limited to professionally created content (37). When evaluating the content of the comments of videos in terms of “likes” videos with “likes” higher than the mean much more frequently discussed HPV-associated cancer, age at which to be vaccinated, asked more questions about vaccination and related costs and insurance coverage suggesting that these are important topics for which information is being sought. Furthermore, it could be implied that the content of the more liked videos were better at communicating this key information, resulting in the greater number of “likes”. Interestingly, there was a high proportion of discussion posts centered around alternative medicine for HPV. Researchers point out that the relationship between complementary and alternative medicine and immunization is intricate (43, 44). Further research is warranted on the content of comments and how influential they may be on vaccination behavior. Collectively, these points serve as a reminder that commentary linked to a post can be framed in a way that is supportive of or inconsistent with the original video. Further, as highly driven by the lay public, comments can shift focus toward and away from the original topic despite being created by a professional.

This study is limited by several factors. Health professionals' status was self-reported and could not be verified. Content creators who worked in the health profession indicated their role on their page profile or conveyed it orally in their videos. As with all cross-sectional studies, the findings cannot be generalized. This is especially true with studies of social media as the content changes constantly and can influence the most popular hashtag, number of videos, number of comments, etc. Limiting the inclusion of videos to those in the English language limits the potential findings. Further, nuances in language choices and interpretation of sentiment can impact coding. For example, prevention tended to be specifically mentioned less in this sample, but could have been implied through non-exact terminology.

The power of provider information is paramount with HPV vaccine uptake (45, 46). With an unprecedented uptick in use of social media, professionals have taken to using these platforms to encourage health behaviors such as receipt of the HPV vaccine. A study of Twitter suggested that messages regarding HPV vaccination are often intended for parents (45). However, more targeted messages toward adolescents may be warranted particularly on platforms such as TikTok which are highly driven by younger audiences. Regardless of the platform, it is important that health professionals who are creating content remain aware of the potential for opposing or non-factual discourse (47). As our findings point out and are reiterated by others, there is a great deal to be learned by assessing open ended conversations (48).

## Data Availability Statement

The original contributions presented in the study are included in the article/supplementary material, further inquiries can be directed to the corresponding author/s.

## Author Contributions

CB and EJ conceptualized the study. EJ collected the data. GH conducted the data analysis. All authors contributed to the manuscript production.

## Conflict of Interest

The authors declare that the research was conducted in the absence of any commercial or financial relationships that could be construed as a potential conflict of interest.

## Publisher's Note

All claims expressed in this article are solely those of the authors and do not necessarily represent those of their affiliated organizations, or those of the publisher, the editors and the reviewers. Any product that may be evaluated in this article, or claim that may be made by its manufacturer, is not guaranteed or endorsed by the publisher.
